# Integrating Pharmacogenomics and Network Topology for Machine Learning Prediction of HLA-Associated Severe Cutaneous Adverse Drug Reactions

**DOI:** 10.3390/ijms27104187

**Published:** 2026-05-08

**Authors:** Tanaporn Ponduan, Arisara Kunsombut, Thummarat Paklao, Apichat Suratanee, Natapol Pornputtapong, Kitiporn Plaimas

**Affiliations:** 1 Department of Mathematics and Computer Science, Faculty of Science, Chulalongkorn University, Bangkok 10330, Thailand; 6880035820@student.chula.ac.th (T.P.); arisaraksb@gmail.com (A.K.); thu2marat@gmail.com (T.P.); 2 Department of Mathematics, Faculty of Applied Science, King Mongkut’s University of Technology North Bangkok, Bangkok 10800, Thailand; apichat.s@sci.kmutnb.ac.th; 3 Intelligent and Nonlinear Dynamic Innovations Research Center, Science and Technology Research Institute, King Mongkut’s University of Technology North Bangkok, Bangkok 10800, Thailand; 4 Department of Biochemistry and Microbiology, Faculty of Pharmaceutical Sciences, Chulalongkorn University, Bangkok 10330, Thailand; 5 Centre of Excellence in Mathematics, Ministry of Higher Education, Science, Research, and Innovation, National University of Sciences, Bangkok 10400, Thailand

**Keywords:** adverse drug reactions, drug–drug interaction network, drug–symptom interaction network, human leukocyte antigen, Extreme Gradient Boosting (XGBoost) model

## Abstract

Adverse drug reactions (ADRs) remain a major clinical challenge and a leading cause of morbidity and mortality worldwide. Among them, severe cutaneous adverse drug reactions (SCARs), including Stevens–Johnson syndrome (SJS) and toxic epidermal necrolysis (TEN), represent life-threatening immune-mediated hypersensitivity responses strongly associated with specific human leukocyte antigen (HLA) alleles. Despite well-established pharmacogenetic associations, current diagnostic strategies remain largely retrospective and lack predictive capability for novel drug–HLA risk pairs. Here, we present an integrative network-informed machine learning framework for predicting HLA-associated SCAR risk by combining pharmacogenomic features, drug chemical structure, and topological descriptors derived from drug–drug and drug–symptom interaction networks. An Extreme Gradient Boosting (XGBoost) classifier trained on integrated HLA allele and drug features, labeled using curated HLA–SCAR associations, achieved an accuracy of 0.860 ± 0.005, an F1-score of 0.689 ± 0.010, with an area under the receiver operating characteristic curve (AUROC) of 0.922 ± 0.003 and an area under the precision–recall curve (AUPRC) of 0.768 ± 0.007. Notably, several predicted positive associations absent from the training data corresponded to biologically plausible and literature-supported cases, including carbamazepine—HLA-B*15:11, supporting the model’s ability to generalize beyond known associations. Molecular docking provides structural evidence for the predicted associations, highlighting allele-specific binding patterns underlying these results. Overall, our results demonstrate that network-informed machine learning provides a proactive and integrative approach to SCAR risk prediction and may support early risk stratification and personalized drug safety assessment in precision medicine.

## 1. Introduction

Adverse drug reactions (ADRs) are harmful or unintended responses to medications administered at standard dosages for treatment, diagnosis, or disease prevention. They range from mild effects, such as nausea or dizziness, to severe outcomes, including life-threatening anaphylaxis or organ damage. ADRs can arise from multiple factors, including drug–drug interactions, patient-specific characteristics (e.g., age, genetics, or underlying conditions), and inappropriate medication use. Effective monitoring and reporting are essential to ensure patient safety and improve therapeutic outcomes. ADRs are broadly classified as Type A or Type B reactions [1]. Type A (augmented) reactions result from the known pharmacological properties of a drug, are dose-dependent, and account for 85–90% of all ADRs [2]. Type B (bizarre) reactions are unpredictable, not related to pharmacological action, and further divided into several subcategories [1]. This work focuses on Type IV hypersensitivity, a delayed immune-mediated reaction involving T lymphocyte activation. Typically occurring 48–72 h after drug exposure, these reactions can cause severe drug-induced skin conditions such as Stevens–Johnson syndrome (SJS) and toxic epidermal necrolysis (TEN) [3], with mortality rates of 10–30% or higher. The severity and complexity of Type IV hypersensitivity highlight the urgent need for improved tools to detect, predict, and mitigate ADRs effectively. Identifying high-risk individuals in advance could reduce the incidence of severe reactions and improve patient outcomes, underscoring the importance of better diagnostic and predictive approaches.

Adverse drug reactions are driven by molecular recognition mechanisms, involving complex interactions between drug metabolites, human leukocyte antigen (HLA) molecules, and T-cell receptors (TCRs) [4]. Genetic factors play an important role in these reactions, highlighting the need for pharmacogenetic testing to identify individuals at higher risk and enabling better-targeted interventions to reduce severe ADRs. However, the early detection and prevention of ADRs remain a major challenge in modern healthcare, with substantial implications for patient safety and healthcare costs. Traditional pharmacovigilance methods, such as spontaneous reporting systems (SRS), electronic health records (EHRs), and wet-lab experiments, are limited by underreporting, delays, and incomplete coverage [5,6].

To overcome these challenges, machine learning (ML) methods have been applied to predict ADRs and drug-induced toxicity, enabling rapid, comprehensive, and precise predictions even before drug synthesis or clinical trials [7]. Among ML approaches, Extreme Gradient Boosting (XGBoost) models have demonstrated strong predictive performance due to their ability to model complex, non-linear relationships and effectively handle heterogeneous and high-dimensional data. Compared to logistic regression, XGBoost does not assume linear relationships and can capture intricate interactions between HLA features, drug structures, and network-derived descriptors. Compared to support vector machines (SVM), it scales more efficiently to large datasets and provides interpretable feature importance. While other ensemble methods such as Random Forest and LightGBM have also been widely used, XGBoost incorporates regularization mechanisms that reduce overfitting and improve generalization performance. These properties make XGBoost particularly suitable for integrating diverse biomedical features and handling imbalanced datasets in SCAR prediction tasks. Previous studies have shown that ML-based models can outperform traditional clinical scoring systems in predicting severe ADR outcomes, such as mortality risk in SJS/TEN patients [8]. In addition, Random Forest-based approaches have been successfully applied to drug-induced gene expression data, identifying predictive gene signatures associated with multiple side effects while addressing data imbalance through techniques such as Synthetic Minority Over-sampling Technique (SMOTE) [2]. These findings highlight the effectiveness of tree-based ensemble methods, which motivates the use of XGBoost in this study. Based on these advantages, XGBoost was selected as the primary modeling approach in this study.

While ML methods improve prediction, they remain challenged by limited flexibility, sparse datasets, and difficulty in detecting rare ADRs [9]. Network analysis, defined as the graph-based modeling of among biological and pharmacological entities (e.g., drugs, proteins, and symptoms), has emerged as a complementary approach. In such frameworks, nodes represent entities and edges represent their interactions, enabling the use of topological features (e.g., degree, centrality, and connectivity patterns) to capture system-level relationships. Several studies [10,11,12,13] have successfully employed network-based approaches to uncover disease-related drugs and biomolecular associations across diverse contexts, from heterogeneous protein networks to immune-related protein interaction networks for drug repurposing. These approaches capture complex topological patterns among biological entities and enhance predictive power. In such networks, drugs, genes, proteins, and symptoms are represented as nodes and their interactions as edges, enabling the modeling of complex relationships [9,14]. Centrality measures provide complementary insights into network structure. Degree centrality reflects immediate ADR risk through direct associations, while eigenvector centrality captures systemic influence across the network. Zhou and Uddin (2023) [15] constructed a drug–drug network from non-clinical sources, identifying weighted degree centrality and weighted PageRank as key predictive features, achieving an AUROC of 0.8210 across tested ADRs [15]. Although machine learning and network-based approaches have shown promise in SCAR risk prediction, existing models have several important limitations. First, many pharmacogenetic studies rely solely on HLA typing, without integrating complementary biological or clinical context, which restricts their ability to capture complex drug–immune system interactions. Second, most approaches do not incorporate network-based information, such as drug–drug or drug–symptom relationships, thereby overlooking higher-order topological patterns that may contribute to adverse reactions. Third, these models often exhibit limited generalizability when applied to unseen drugs or populations. Finally, current methods struggle to accurately predict rare drug–HLA associations due to data sparsity and class imbalance. In addition, most existing studies focus on population-level side effects or generic drug–target interactions, without explicitly modeling HLA-mediated hypersensitivity mechanisms. The integration of pharmacogenomic data with biological network topology therefore remains largely underexplored, particularly in the context of severe cutaneous adverse drug reactions (SCARs). These limitations highlight the need for integrative approaches that combine pharmacogenomic, chemical, and network-level information to improve predictive performance and robustness.

To address these gaps, we propose an integrative framework that combines HLA sequence features, drug chemical descriptors, and topological measures derived from drug–drug and drug–symptom interaction networks within an XGBoost learning paradigm. By systematically evaluating the contribution of network-derived features and validating selected predictions through molecular docking, our study advances predictive modeling of HLA-associated SCAR risk while providing mechanistic insights into allele-specific drug hypersensitivity. Collectively, our contributions are threefold: (i) construction of an integrated pharmacogenomic–network dataset for HLA-associated SCARs, (ii) development of a network-informed XGBoost model for SCAR risk prediction, and (iii) structural validation of model predictions using molecular docking.

## 2. Results

### 2.1. Overall Workflow of the Proposed Framework

**Figure 1:**
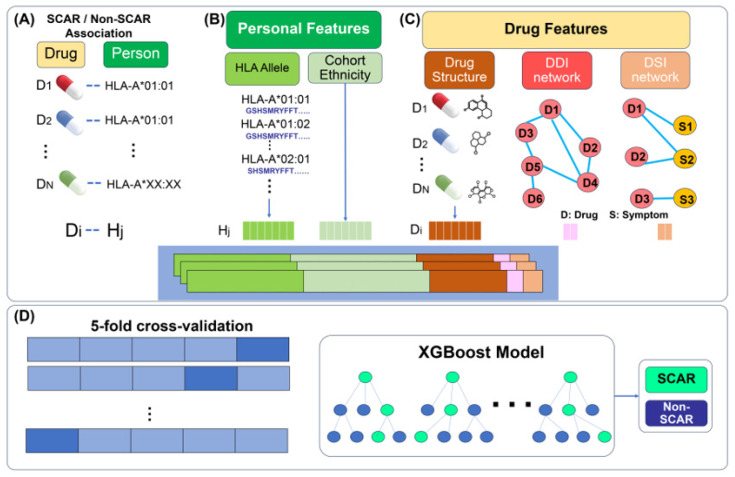
Schematic overview of the proposed framework for adverse drug reaction (ADR) prediction. The framework integrates three input datasets: (**A**) the HLA-ADR dataset consisting of drug–HLA associations; (**B**) personal features, including HLA allele protein sequences and cohort ethnicity. (**C**) drug features, comprising drug structures and topological features derived from DDI and DSI networks. Features extracted from these datasets are encoded and concatenated to form unified representations. (**D**) The learning and evaluation pipeline, including dataset splitting, five-fold cross-validation, XGBoost model training, and performance assessment.

The overall workflow of the proposed framework is illustrated in Figure 1. To build a classification model for SCAR rick prediction, we first extract features for all pairs of drugs and HLA alleles as shown in Figure 1A–C. Figure 1A illustrates the HLA–ADR dataset, which consists of drug–HLA associations. Figure 1B represents personal features, including HLA allele genotypes and cohort ethnicity. Figure 1C illustrates drug-related features, comprising drug chemical structures as well as topological features derived from the drug–drug interaction (DDI) and drug–symptom interaction (DSI) networks. Features extracted from these datasets are encoded into numerical vectors and subsequently concatenated to form unified representations. The number of concatenated feature vectors corresponds to the number of samples, which is equal to the number of HLA–ADR association records. The learning and evaluation process of the proposed framework is illustrated in Figure 1D, including dataset splitting, five-fold cross-validation, XGBoost model training, and performance assessment.

To evaluate the performance of the proposed framework, the dataset is first divided into training and independent testing subsets. Five-fold cross-validation is then applied to further split the training dataset into training and validation subsets. The training data are used to train an XGBoost classifier for SCAR risk prediction, while the testing data are used to assess the generalization ability and predictive performance of the model.

### 2.2. Data Characteristics and Exploratory Analysis

To characterize the integrated dataset used for model development, we first summarized the constituent data sources and feature categories (Table 1). The dataset comprises two feature categories: personal features and drug features. Personal features include HLA protein sequence-derived features and cohort ethnicity information, while drug features consist of chemical structure descriptors and network-derived topological features from DDI and DSI networks (see Section 4 for more detail). Together, these heterogeneous data types capture genetic susceptibility, molecular structure, demographic context, and systemic pharmacological behavior relevant to adverse drug reactions. In addition, where applicable, we examined the statistical distributions of individual features as well as their potential associations with ADR outcomes, including correlation analyses and univariate exploratory assessments, to gain insights into feature relevance and data characteristics prior to model training.

After data integration and preprocessing, the dataset included 116 distinct HLA alleles, 15 drugs, and 4 ethnicity categories (Black, Caucasian, Diverse, and Mongoloid). In addition, 4 network-derived features were constructed from DDI and DSI networks. The full dataset comprised 825 instances and 361 feature columns. Prior to feature selection, the feature space consisted of 113 HLA sequence-derived features, 237 drug-related features, 4 ethnicity features, and 4 network-based features. Subsequently, feature selection was performed to reduce dimensionality, and the final model was trained using the top 100 selected features. The final dataset consists of 825 instances, each representing a unique drug–allele–population association. The binary target variable distinguishes between SCAR-associated (positive) and non-SCAR-associated (negative) drug–HLA pairs. The negative class comprises pairs that do not meet statistical significance criteria or are not reported in curated datasets; therefore, it may include underpowered, unstudied, or potentially positive associations rather than confirmed true negatives. This introduces potential label noise and should be considered when interpreting model performance. As expected in pharmacovigilance data, the class distribution is imbalanced, with 653 non-SCAR-associated cases and 172 SCAR-associated cases, resulting in approximately a 3:1 ratio. This imbalance reflects the relative rarity of severe cutaneous adverse drug reactions in the general population and underscores the importance of using appropriate evaluation metrics and resampling strategies.

Carbamazepine (*n* = 213) and phenytoin (*n* = 139) emerged as the most common causative drugs, consistent with their well-documented associations with HLA-mediated hypersensitivity. The cohort ethnicity distribution was dominated by Mongoloid populations (*n* = 375), reflecting the population structure of available pharmacogenomic studies. The most frequently observed alleles included HLA-B*13:01 (*n* = 18), HLA-B*15:02 (*n* = 17), and HLA-B*15:11 (*n* = 15), all of which have been implicated in severe drug-induced cutaneous reactions in previous studies [16].

Exploratory analysis of the drug–drug interaction network revealed a highly heterogeneous connectivity structure. The eigenvector centrality distribution indicates that only a small subset of drugs occupies influential positions within highly connected subnetworks, while the majority exhibit low global influence. Similarly, the degree distribution (see Supplementary File S1) shows a non-uniform pattern, with some drugs exhibiting a higher number of interactions compared to others. This heterogeneity suggests that certain drugs may play more prominent roles in the pharmacological interaction landscape. Such topological variability supports the inclusion of network-derived features to capture systemic risk patterns beyond individual drug or allele effects.

### 2.3. Feature Importance and Contribution of Network-Based Features

To assess the contribution of network-derived features and to characterize the relative importance of different feature groups, we evaluated XGBoost models under four feature configurations: (i) a baseline model including genetic data, cohort ethnicity, and drug features, excluding all DDI and DSI features; (ii) the baseline model incorporating DSI features only; (iii) the baseline model incorporating DDI features only; and (iv) a model integrating both DDI and DSI features. The baseline feature set was designed to capture the key determinants of SCAR risk, comprising HLA sequence features (genetic variation and peptide-binding specificity), drug chemical descriptors (molecular structure and properties), and ethnicity (population-specific differences in allele frequencies and susceptibility). These components reflect established pharmacogenomic evidence that SCAR risk arises from interactions between host genetics, drug structure, and population background. Model performance was evaluated using AUROC, AUPRC, F1-score, and accuracy, as summarized in Table 2.

The baseline model demonstrated robust performance (AUROC = 0.88853 ± 0.03828), indicating that HLA sequence features, drug chemical descriptors, and demographic variables capture substantial predictive signals. Incorporation of DSI and DDI features increased AUROC from 0.88853 to 0.88989 and AUPRC from 0.68953 to 0.69185, along with improvements in F1-score (from 0.58571 to 0.59155) and accuracy (from 0.83600 to 0.83939), suggesting that network topology provides complementary information beyond molecular and genetic features. However, models trained overly combined feature did not show substantial performance improvement and instead exhibited comparable or only marginally different results across evaluation matric. These findings highlight that selective integration of biologically meaningful features is more effective than indiscriminate feature aggregation.

When considering AUPRC performance, the incorporation of DDI or DSI features resulted in a slight reduction in AUPRC despite improvements in AUROC. This discrepancy suggests that while network-derived features enhance overall discriminative ability, they may also increase false positive rates in the minority class, which is critical under imbalanced data conditions. Since AUPRC is more sensitive to class imbalance and precision–recall trade-offs, the observed decrease indicates that the inclusion of network features may introduce noise or dilute class-specific signals, thereby reducing precision. These results imply that although DDI and DSI features contribute complementary global information, their integration requires careful feature selection and calibration.

**Figure 2:**
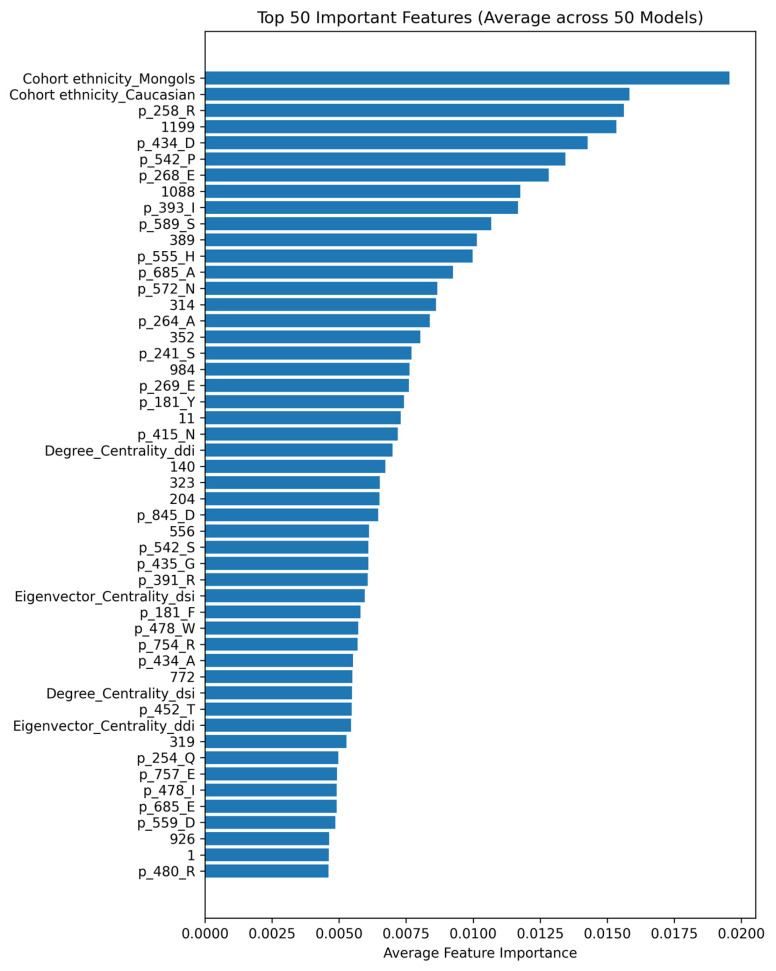
Top 50 most important features identified by the XGBoost model based on the mean feature importance scores averaged across all cross-validation runs. These scores represent the relative contribution of each feature to the model’s decision-making process.

**Figure 3:**
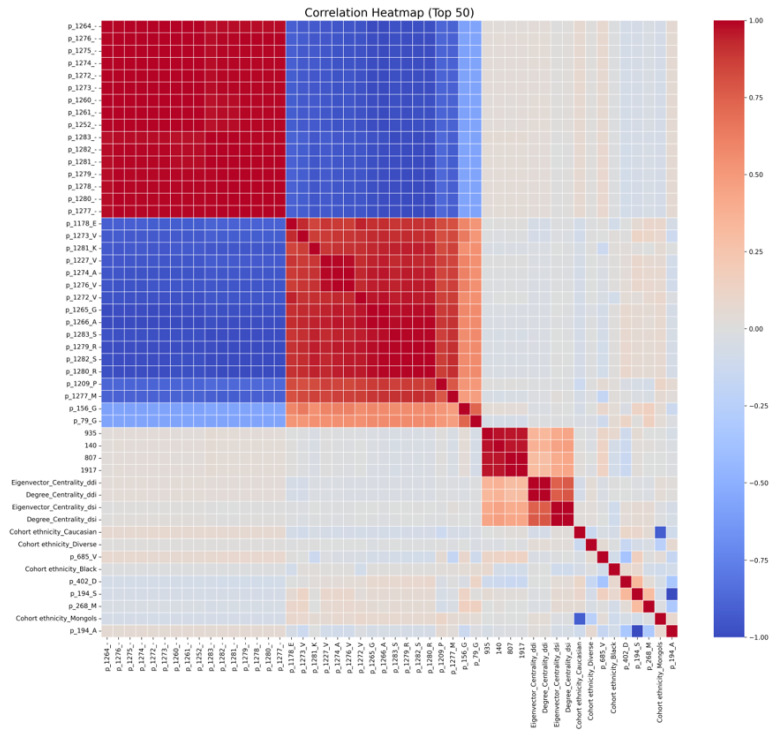
Correlation heatmaps of features selected by the XGBoost model. The top 50 features selected by the XGBoost capture the most informative biological signals while reducing redundancy from the full feature set, thereby enhancing interpretability and model performance.

Feature importance was analyzed by averaging results across cross-validation runs to obtain a stable ranking of predictive features. The list of top 100 important features is provided in Supplementary Table S1. Figure 2 and Figure 3 represent the top 50 features ranked based on their importance scores and their correlation heatmap, respectively. The correlation heatmap in Figure 3 further illustrates the pairwise relationships among the top 50 features, revealing generally low-to-moderate correlations across most features, which indicates limited multicollinearity and supports the robustness of the selected feature set. Feature importance analysis suggests that a subset of features contributes most to the predictive performance, indicating potential redundancy among less informative features. This demonstrates suggesting reduced redundancy and improved interpretability of the feature space compared to the full feature set, indicating that a compact subset of informative features is sufficient to capture key biological patterns.

The top-ranked features included HLA sequence positions, drug chemical descriptors, and network centrality measures, reflecting the multi-modal nature of the predictive signal. Cohort ethnicity also appeared among influential features, suggesting that population-specific genetic backgrounds contribute to susceptibility.

The feature importance analysis (Figure 2) highlights several position-specific HLA sequence features (e.g., p_258_R, p_242_P, and p_434_D) as major contributors to model predictions, indicating that variation at specific amino acid residues plays a critical role in SCAR risk. These positions are likely associated with residues located within or proximal to the α1 and α2 domains of the HLA molecule, which form the peptide-binding groove. Variations in these regions can alter the physicochemical properties and geometry of key binding pockets, thereby influencing peptide repertoire, drug binding affinity, or the formation of drug–peptide–HLA complexes. Such structural changes are consistent with known mechanisms of HLA-associated hypersensitivity, where specific residues modulate antigen presentation and T-cell activation. The prominence of these position-specific features suggests that the model captures functionally important hotspots within the HLA molecule that govern immune recognition. In parallel, molecular fingerprints provide complementary information by encoding drug-specific chemical substructures, supporting a mechanism in which SCAR risk arises from the interaction between drug molecular properties and critical HLA binding residues.

In addition to HLA sequence features, drug-related molecular descriptors derived from Morgan fingerprint encoding constituted a substantial portion of highly ranked predictors. Several network-derived metrics from DDI and DSI networks were consistently observed among the top features. Topological measures such as degree centrality and eigenvector centrality captured connectivity and influence within interaction networks. These results support the role of network topology as a complementary source of biological information.

Figure 3 presents the correlation heatmap of the top 50 features, revealing a clear block-structured pattern. Two prominent clusters are observed among the HLA sequence-derived features. Within each cluster, features are highly positively correlated (red blocks), while strong negative correlations (blue regions) are evident between the two clusters. This pattern likely reflects mutually exclusive or compensatory amino acid variations across aligned HLA positions, indicating redundancy within groups but discriminative contrast between groups. In contrast, drug-related features (e.g., molecular fingerprint components such as p_79_G, p_93_S, p_140, p_807, and p_1917) show moderate intra-group correlations but relatively weak associations with HLA sequence features. This suggests that drug descriptors provide complementary, largely independent information to the genetic features, supporting their combined use in the predictive model. Network-derived features, including degree and eigenvector centrality from DDI and DSI networks, form a smaller correlated cluster. Their moderate correlations indicate shared topological properties while still contributing non-redundant information. Similarly, cohort ethnicity variables exhibit expected correlations within demographic categories but remain largely independent from molecular and network features. Overall, the heatmap indicates that while certain groups of features (particularly HLA sequence features) exhibit redundancy, the model benefits from integrating heterogeneous feature types—genetic, chemical, network, and demographic—which are largely complementary. This diversity likely contributes to the model’s robust performance, although the presence of highly correlated features suggests that further dimensionality reduction or feature selection could improve model interpretability and potentially reduce overfitting.

The feature importance analysis indicates that a subset of features contributes most strongly to model predictions. To evaluate whether the feature space could be reduced without compromising performance, we conducted additional analyses using progressively smaller subsets of top-ranked features. While reduced feature sets preserved most of the predictive performance, inclusion of lower-ranked features provided complementary information that modestly improved model robustness and generalization. Accordingly, the full feature set was retained in the final model.

### 2.4. Model Performance and Validation

**Figure 4:**
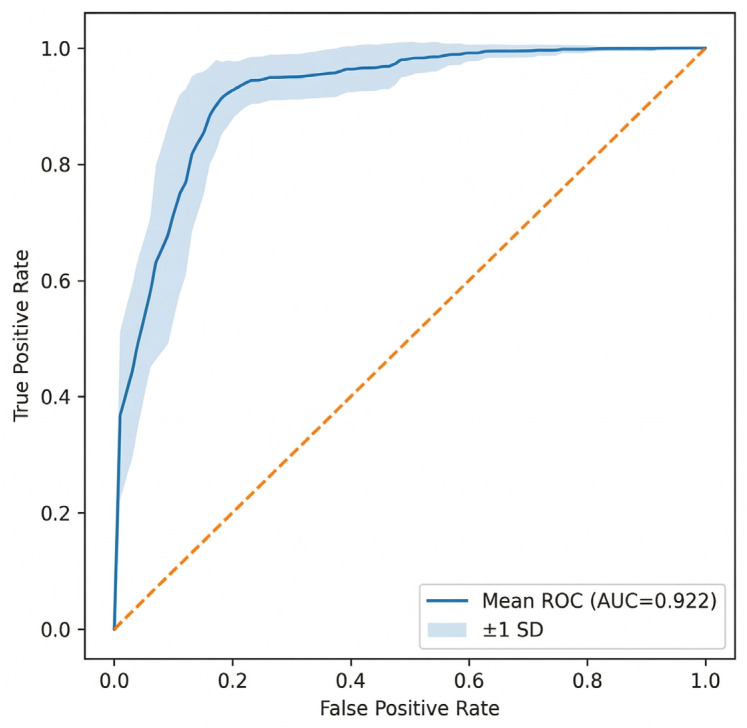
Receiver Operating Characteristic (ROC) curve showing the performance of the XGBoost model for predicting ADR-associated drug–allele interactions. The blue curve represents the mean ROC obtained from repeated cross-validation, with the shaded region indicating the standard deviation across folds. The model achieved a mean AUROC of 0.922 ± 0.035, demonstrating consistent discriminative ability compared with the XGBoost baseline (dashed line).

**Figure 5:**
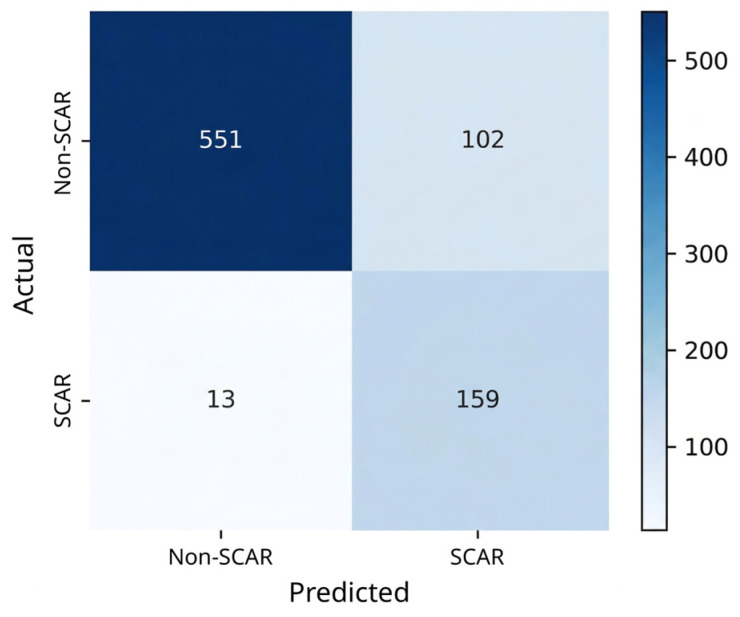
The confusion matrix of classification model. The heatmap displays the number of actual positive and negative instances on the *y*-axis and predicted negative and positive instances on the *x*-axis. The intensity of the blue color indicates the frequency of samples in each category, with darker shades representing higher counts.

The XGBoost classifier was optimized using Optuna 4.8.0 with a total of 25 trials and a nested cross-validation framework. The hyperparameter search space included number of estimators (100–500), maximum depth (3–10), learning rate (0.01–0.3), subsample ratio (0.6–1.0), column subsampling ratio (0.6–1.0), and gamma (0–5). The final model used the best parameter set selected from the optimization procedure. All models were trained with a fixed random seed (random state = 42) to ensure reproducibility. The predictive performance of the proposed model was evaluated using multiple classification metrics under a nested stratified 5-fold cross-validation framework. In each iteration, the dataset was split into 80% training and 20% testing sets using stratified sampling to preserve the class distribution of SCAR and non-SCAR samples. The test set in each fold was kept completely independent and was not used during model training or optimization. Within the training set, hyperparameter tuning was performed using Optuna 4.8.0 with stratified cross-validation, and SMOTE was applied only to the training data after the split to address class imbalance while preventing data leakage (see Section 4). The final model achieved an accuracy of 0.860 ± 0.005, indicating stable performance across repeated cross-validation experiments. For SCAR prediction (positive class), the model obtained an F1-score of 0.689 ± 0.010. In addition, the model achieved an AUROC of 0.922 ± 0.003 (in Figure 4), and an AUPRC of 0.768 ± 0.007, demonstrating strong discriminative ability and precision under class imbalance. To assess potential overfitting, model performance was evaluated across training and test folds within the nested cross-validation framework. The comparable performance between training and test evaluations indicates that the model did not exhibit significant overfitting and demonstrates good generalization ability. To determine an appropriate decision threshold, Youden’s J statistic was applied to the receiver operating characteristic (ROC) curve. The optimal threshold at 0.1505 maximized the difference between true positive and false positive rates. At this threshold, the model achieved an accuracy of 0.8606 and an F1-score of 0.735. The confusion matrix (in Figure 5) showed correct classification of 551 non-SCAR-associated cases and 159 ADR cases, with 102 false positives (see Supplementary Table S2). To reduce false positives and improve specificity, future work will incorporate structure-informed features, refine negative labels, integrate allele grouping information, and apply calibration or cost-sensitive learning.

Importantly, analysis of false-positive predictions revealed biologically plausible drug–allele associations, particularly for carbamazepine and phenytoin. Some of these associations were absent from the training data but supported by independent studies, suggesting the model captures latent biological patterns.

Further examination showed that many false positives involve clinically relevant drugs and HLA alleles with known or closely related SCAR risk. In particular, predictions are enriched for aromatic anticonvulsants (e.g., carbamazepine, phenytoin, lamotrigine, and oxcarbazepine) and allopurinol, which are well-established triggers of SCAR. Several predicted pairs include alleles such as HLA-B*15:02, HLA-A*31:01, and HLA-B*58:01, as well as closely related variants within the same HLA families (e.g., B15 subtypes or A02 variants). This pattern suggests that a subset of the “false positives” may reflect shared structural or functional properties within HLA supertypes or class effects of chemically similar drugs that are not fully captured by current labels. The clustering of predictions within B15 and A02 groups further indicates that the model is sensitive to sequence-level similarities in peptide-binding regions, potentially generalizing risk signals across related alleles. Notably, one of the false-positive predictions—carbamazepine with HLA-B*15:11—has been independently confirmed to be associated with severe cutaneous adverse drug reactions, including Stevens–Johnson syndrome (SJS) and toxic epidermal necrolysis (TEN), in non–HLA-B*15:02 carriers [17,18]. This concordance between model prediction and external clinical evidence supports the biological validity of the framework and highlights its potential utility for uncovering previously unannotated high-risk HLA–drug associations. To further assess the structural plausibility of selected predictions, molecular docking analyses were subsequently performed, as described in the following subsection.

### 2.5. Docking-Based Structural Validation

**Figure 6:**
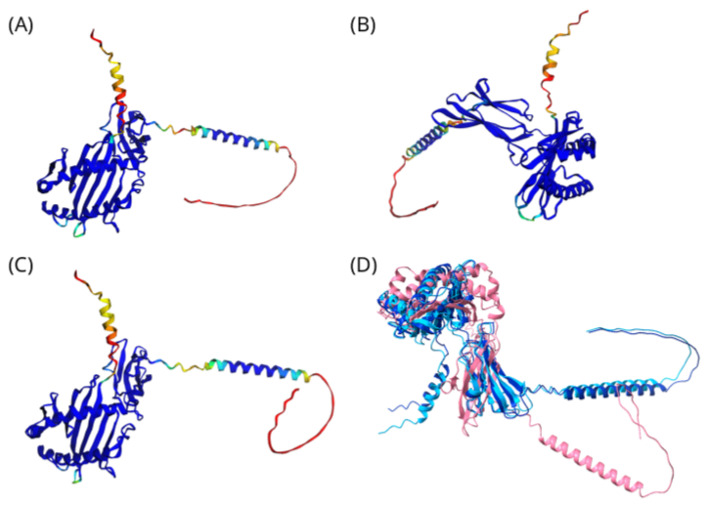
AlphaFold2-predicted structures of representative HLA class I alleles. (**A**) HLA-B*15:02, (**B**) *HLA-B15:11*, and (**C**) HLA-C*44:03. Structures in panels A–C are colored according to AlphaFold2 predicted local confidence (pLDDT) scores, where dark blue indicates high-confidence regions, cyan/green indicates intermediate confidence, and yellow-to-red indicates lower-confidence or flexible regions. (**D**) Structural superposition of the three alleles shows strong conservation of the HLA class I fold, particularly in the α1 and α2 domains forming the peptide-binding groove. HLA-B*15:11 is shown in dark blue, HLA-B*15:02 in sky blue, and HLA-C*44:03 in pink. Subtle differences in loop regions and flexible termini may contribute to allele-specific drug-binding properties and hypersensitivity risk.

To further assess the biological plausibility of selected model predictions, molecular docking analyses were performed using the updated SwissDock web server, which integrates the Attracting Cavities approach and AutoDock Vina [19] to examine potential interactions between carbamazepine and representative HLA class I alleles. Three alleles were examined: the well-established risk allele HLA-B*15:02, the predicted risk allele HLA-B*15:11, and the non-associated allele HLA-C*44:03 as a negative control. Whenever available, experimentally determined HLA structures from the Protein Data Bank (PDB) were prioritized as templates for structural analysis. In cases where no experimentally resolved structures were available for specific alleles, AlphaFold2-predicted models were used. AlphaFold2-predicted structures of representative HLA class I alleles are shown in Figure 6. Structural comparison reveals a high degree of conservation in the overall HLA class I fold, particularly within the α1 and α2 domains that form the peptide-binding groove, while subtle variations in loop regions and flexible termini may contribute to allele-specific drug-binding properties.

**Figure 7:**
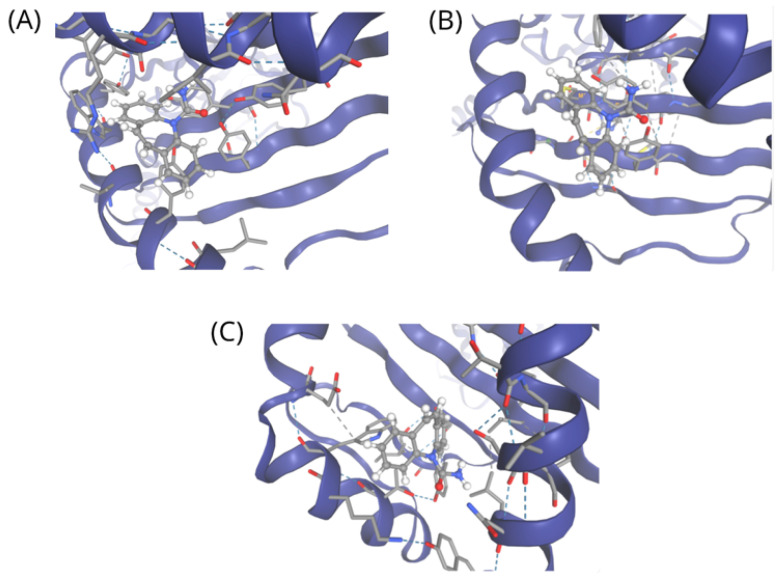
Molecular docking of carbamazepine with representative HLA class I alleles. (**A**) Carbamazepine binds within the peptide-binding groove of the known risk allele HLA-B*15:02. (**B**) A similar binding orientation is observed for the predicted risk allele HLA-B*15:11. (**C**) In contrast, docking to the non-associated allele HLA-C*44:03 shows binding at a distinct site outside key peptide presentation and T-cell receptor-interacting regions. HLA proteins are shown as blue cartoon structures, carbamazepine molecules are represented as gray stick models, and dashed lines indicate predicted intermolecular interactions/hydrogen bonds.

Docking simulations were performed using the updated SwissDock web server, which integrates the Attracting Cavities approach and AutoDock Vina [19]. The docking search space was defined using a cubic box of 20 × 20 × 20 Å centered at coordinates (13.95, −8.11, −7.43), Sampling exhaustivity was set to 90 to ensure sufficient exploration of conformational space, with cavity prioritization set to 70 to favor energetically favorable binding regions. Multiple docking runs were performed using 4 conditions to improve robustness and reproducibility of predicted binding poses. Docking was conducted to provide complementary structural insights into potential drug–HLA interactions, rather than to serve as direct validation of clinical outcomes. Docking results indicate that carbamazepine can adopt stable binding conformations within the peptide-binding groove of HLA-B*15:02, with an Allele Combination (AC) score of 13.86 and a SwissParam score of −6.75, as shown in Figure 7A. A comparable binding profile was observed for HLA-B*15:11 (AC score = 10.79; SwissParam score = −6.99), as shown in Figure 7B. These results suggest a structural interaction profile similar to that of a known risk allele, supporting the model’s predicted positive association and aligning with emerging clinical evidence linking this allele to carbamazepine-induced severe cutaneous adverse drug reactions in non—HLA-B*15:02 carriers.

In contrast, docking to the non-associated allele HLA-C*44:03 yielded interactions that were less biologically relevant despite similar docking scores (AC score = 15.62; SwissParam score = −6.68), as shown in Figure 7C. In this case, carbamazepine did not localize to regions implicated in peptide presentation or T-cell receptor recognition, suggesting that binding context and spatial orientation, rather than docking score alone, are critical determinants of immunogenic potential. Collectively, these results provide complementary structural support for the model’s predictions. Although molecular docking cannot directly predict clinical hypersensitivity reactions, the concordance between network-informed machine learning predictions, allele-specific binding patterns, and independent clinical reports strengthens the biological plausibility and mechanistic relevance of the proposed framework. Importantly, these docking results do not establish clinical risk, instead provide structural insight into potential allele-specific interaction patterns.

Overall, these findings further support the model predictions. While docking does not establish clinical causality, the agreement between machine learning predictions, structural analysis, and literature evidence reinforces the biological plausibility of the proposed framework.

## 3. Discussion

Severe cutaneous adverse drug reactions (SCARs), including Stevens–Johnson syndrome (SJS) and toxic epidermal necrolysis (TEN), represent some of the most devastating outcomes of drug hypersensitivity, with strong genetic associations with specific HLA alleles. Despite substantial advances in pharmacogenomics, most existing approaches remain retrospective, allele-specific, or limited to well-characterized drug–HLA pairs, restricting their utility for proactive risk assessment and the discovery of novel associations. In this study, we present an integrative, network-informed machine learning framework that combines HLA sequence features, drug chemical descriptors, demographic context, and interaction network topology to model HLA-associated SCAR risk within a unified predictive paradigm.

Our results demonstrate that the proposed framework achieves consistent predictive performance, with good recall and a moderate F1-score, indicating effective identification of high-risk drug–allele combinations. Importantly, robust performance was observed even in the baseline model without network derived features, highlighting the substantial predictive value of HLA sequence variation and drug chemical structure. HLA sequence features were encoded using one-hot representations derived from multiple sequence alignment to preserve position-specific information in an interpretable form. However, this approach does not explicitly capture physicochemical or structural properties that may influence HLA–drug interactions. While we partially addressed structural relevance through molecular docking analysis, future work could incorporate physicochemical descriptors and structure-informed features (e.g., derived from AlphaFold2) to better capture biologically meaningful variation and improve model performance. The incorporation of DDI and DSI networks feature increased model performance, with AUROC improving from 0.88853 to 0.88989 and AUPRC from 0.68953 to 0.69185, indicating that network topology provides complementary systemic context beyond molecular and genetic features. This finding aligns with the notion that adverse drug reactions arise not only from direct molecular recognition but also from broader pharmacological and phenotypic interaction landscapes. Notably, DSI features appear to provide more informative signals than DDI features. This may be attributed to the fact that DSI networks capture phenotypic manifestations of drug responses, which are more directly related to clinical outcomes such as SCARs. In contrast, DDI networks primarily reflect co-administration patterns and pharmacokinetic interactions, which may not directly correspond to immune-mediated hypersensitivity mechanisms. Since SCARs are driven by immune recognition processes involving HLA presentation and downstream T-cell activation, symptom-level associations may better reflect the underlying pathophysiological context than drug co-exposure patterns. This suggests that integrating phenotype-oriented network features provides a more biologically relevant representation for predicting severe adverse drug reactions. A key insight from our feature importance and top-*k* analyses is that predictive performance saturates at a relatively small subset of informative features. The feature importance analysis indicates that a reduced subset of features may achieve comparable performance, suggesting the potential for dimensionality reduction without substantial loss of predictive accuracy. This may improve model efficiency and interpretability in future work, with an elbow point observed around 100 features. This suggests that SCAR risk is driven by a compact set of biologically meaningful signals rather than diffuse contributions from high-dimensional noise. Such compactness enhances model interpretability and reduces the risk of overfitting, which is particularly important in pharmacogenomic applications where sample sizes are inherently limited.

Notably, examination of predicted positive associations absents from the training data revealed that several such drug–allele pairs correspond to biologically plausible and independently reported associations. In particular, the prediction of carbamazepine–HLA-B*15:11, subsequently supported by external clinical evidence, highlights the model’s capacity to uncover latent risk patterns beyond known annotations. This ability to generalize beyond curated databases represents a key advantage over traditional rule-based or allele-specific screening approaches and suggests potential utility for hypothesis generation and early safety signal detection. In comparison with previously reported adverse drug reaction prediction models, such as the study by Liang et al. (2019) [20], which employs multi-view learning to integrate chemical, biological, and phenotypic features, the proposed framework extends this approach by incorporating HLA sequence features and network-derived descriptors. While Liang et al. focused on general drug side-effect prediction, our model specifically targets HLA-associated SCARs, a more complex and clinically critical subset of immune-mediated adverse drug reactions.

Furthermore, our model achieves an AUROC of 0.922 and an F1-score of 0.689, demonstrating competitive performance compared to previously reported models (AUROC ~0.80–0.88), while providing improved capability to identify allele-specific risk patterns. These results highlight the advantage of integrating pharmacogenomic, chemical, and network-level information within a unified predictive framework.

The structural docking analyses provide supporting structural plausibility for these findings, with particular emphasis on the spatial context of ligand binding rather than docking scores alone. The observation that carbamazepine exhibits favorable binding within the peptide-binding grooves of both HLA-B*15:02 and HLA-B*15:11, but not in immunologically relevant regions of the non-associated allele HLA-C*44:03, supports an allele-specific mechanistic basis for the predicted associations. While molecular docking alone is insufficient to directly infer clinical hypersensitivity reactions, the alignment of machine learning predictions, allele-specific interaction profiles, and independent clinical observations provides convergent support for the biological plausibility of the proposed framework.

From a methodological perspective, this study illustrates the value of integrating multi-modal data sources—genetic, chemical, demographic, and network-based—within a single predictive model. While network features contributed only relatively modest performance improvements in this dataset, their inclusion provides important systems-level context and may become increasingly valuable as interaction databases expand in coverage and resolution. The observed scale-free topology of the drug–drug interaction network further supports the relevance of topological descriptors in capturing systemic pharmacological risk patterns. The relatively limited contribution of network-derived features may also reflect the fact that current interaction networks do not fully capture the complex immunological mechanisms underlying HLA-mediated hypersensitivity reactions. Although the observed improvement in predictive performance from network-derived features is modest, these features provide complementary system-level information that is not captured by molecular or genetic descriptors alone. In particular, drug–symptom and drug–drug interaction networks encode higher-order pharmacological and phenotypic relationships that may contribute to adverse drug reaction risk in a non-linear and indirect manner. Therefore, their value lies not only in immediate performance gain but also in enhancing biological interpretability and capturing mechanistic context.

The presence of false-positive predictions suggests that while the model captures latent biological signals, some noise and overgeneralization remain, particularly in cases with limited training representation. Future improvements could include incorporating more refined HLA–peptide structural information, integrating graph-based embedding methods for network features, and applying calibrated probability thresholds to improve decision boundaries. Additionally, expanding training data with more diverse populations may further reduce spurious associations and improve model specificity.

In addition, the contribution of ethnicity-related features should be interpreted with caution. While these variables may capture population-level differences in HLA allele frequencies and drug response patterns, they may also reflect underlying population structure rather than direct causal biological mechanisms. As a result, the inclusion of ethnicity features may introduce potential confounding effects, which could limit the generalizability of the model when applied to populations with different genetic backgrounds. Future studies should explore strategies to disentangle population-specific effects from true biological signals, such as incorporating more diverse cohorts or using ancestry-informed modeling approaches.

Several limitations should be acknowledged. First, the dataset size is constrained by the availability of curated HLA–ADR associations, and the population distribution is skewed toward specific ethnic groups, potentially limiting generalizability. Second, the use of centrality-based network features provides a relatively coarse representation of interaction topology; more expressive approaches, such as graph embeddings, may capture higher-order structure and improve predictive performance. Third, although SMOTE [2] was applied to mitigate class imbalance, synthetic sampling may introduce biologically implausible patterns and should be interpreted with caution. Furthermore, the use of Fisher’s exact test for label construction may lead the model to partially recapitulate existing statistical associations, potentially limiting its ability to discover novel biological relationships and affecting generalizability to unseen drug–HLA pairs.

Finally, molecular docking provides only indirect structural support and does not capture the full complexity of immune-mediated hypersensitivity reactions. In particular, the current approach does not consider the role of antigenic peptides, T-cell receptor (TCR) recognition, or the cellular microenvironment, all of which are critical components of HLA-mediated immune activation. In addition, docking simulations are inherently static and do not account for dynamic conformational changes, peptide loading processes, or downstream signaling events. Therefore, while docking results provide useful structural insights, they should be interpreted as supportive rather than definitive evidence of clinical risk.

Despite these limitations, the proposed framework represents a meaningful step toward proactive, data-driven assessment of HLA-associated SCAR risk. By integrating pharmacogenomics with network biology and structural validation, this approach moves beyond allele-specific screening toward a more generalizable and discovery-oriented paradigm. In clinical settings, such a framework may support early risk stratification, guide safer drug selection, and prioritize drug–allele pairs for experimental validation. More broadly, the integrative strategy presented here is extensible to other immune-mediated adverse drug reactions and complex pharmacogenomic phenotypes.

## 4. Materials and Methods

### 4.1. Dataset and Preparation

**Figure 8:**
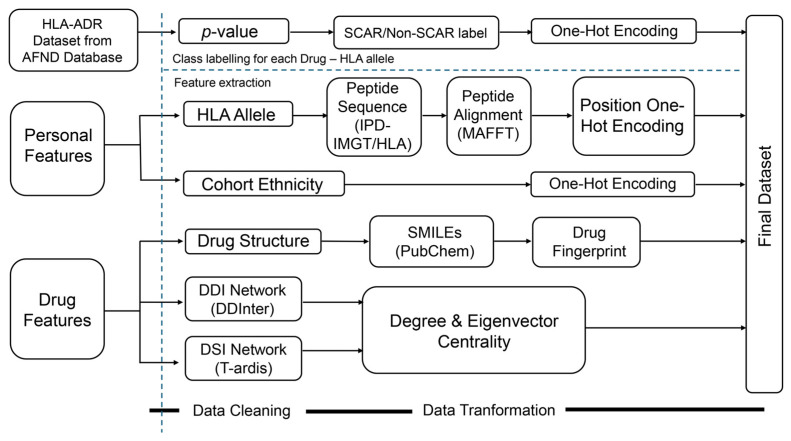
Overview of the data processing and feature integration workflow. Drug–HLA records from AFND [16] were filtered to extract drug names, HLA alleles, and cohort ethnicity. The cohort ethnicity variable was subsequently transformed using one-hot encoding. Drug structures from PubChem [21] were encoded as Morgan fingerprints, while HLA allele sequences from the IPD-IMGT/HLA Database [22] were aligned using Multiple Alignment using Fast Fourier Transform (MAFFT) [23] and transformed into position-specific one-hot features. Network features derived from DDI network retrieved from DDInter [24,25] and from DSI network from T-ARDIS [26,27] were incorporated using degree and eigenvector centrality. All features were integrated into a final dataset for machine learning analysis. Dashed blue lines indicate workflow stage separation, while bold black lines at the bottom denote the major processing phases, including data cleaning and data transformation.

Dataset preparation is illustrated in Figure 8. The pipeline begins with the collection of HLA–ADR association records from the Allele Frequency Net Database (AFND) [16], followed by systematic inclusion and exclusion criteria to retain high-confidence drug–allele–population triplets, defined as those with complete and unambiguous information on drug name, HLA allele, and cohort ethnicity, and supported by statistically significant case–control differences (Fisher’s exact test, *p* ≤ 0.05) within population-specific datasets (see Section 4.1.1 for details on data labeling). Two categories of feature sets, namely personal features and drug features, were then extracted for each drug allele pair. The baseline feature set was defined based on domain knowledge and included HLA sequence features and drug chemical descriptors, both of which are directly related to SCAR pathophysiology. Cohort ethnicity was additionally incorporated to capture population-level variation in HLA allele distribution and drug response patterns.

Personal features consisted of HLA allele peptide sequences and cohort ethnicity. The HLA allele peptide sequences were obtained from the IPD-IMGT/HLA database [22] and aligned using MAFFT (v7.526) [23] to ensure positional consistency across alleles. Drug features were obtained separately, including drug chemical structures and network-topology features derived from DDI and DSI networks. All feature types were subjected to data cleaning and transformation before being merged into a unified feature matrix. This final dataset served as the input for XGBoost model training, validation, and performance evaluation.

#### 4.1.1. Data Labeling

HLA–ADR association records were obtained from the Allele Frequency Net Database (AFND, accessed on 7 April 2024) [16]. Data were collected within the available reporting period of the database, and only records with complete information on drug name, HLA allele, and cohort ethnicity were retained. Duplicate entries, ambiguous drug names, and records lacking statistical information were excluded to ensure data quality and reproducibility. Systematic inclusion and exclusion criteria were then applied to identify high-confidence drug–allele–population triplets, defined as those with complete and unambiguous information on drug name, HLA allele, and cohort ethnicity, and supported by statistically significant case–control differences within population-specific datasets. This study specifically focuses on drug-induced severe cutaneous adverse reactions (SCARs), including Fixed Drug Eruption (FDE), Maculopapular exanthema (MPE), Stevens–Johnson syndrome (SJS) and Toxic epidermal necrolysis (TEN), which are life-threatening immune-mediated hypersensitivity responses. Accordingly, only drug–allele associations relevant to SCARs were retained for analysis. A total of 17 clinically relevant drugs selected based on their reported associations in the literature and their availability in the AFND dataset [16]. After filtering for qualified drug–allele pairs, 15 drugs were retained for subsequent machine learning analysis. These drugs include levetiracetam, carbamazepine, piroxicam (as a representative of oxicam NSAIDs), trichloroethylene, valproic acid, phenobarbital, lamotrigine, methazolamide, nevirapine, sulfamethoxazole, paracetamol, aspirin, oxcarbazepine, allopurinol, and phenytoin. All data sources were then integrated into a unified dataset based on drug names and HLA allele identifiers.

To construct a binary classification task for SCAR risk prediction, drug–allele associations were labeled based on statistical comparison of allele frequencies between hypersensitive cases and drug-exposed controls. For each drug–allele pair, allele frequencies were compared using Fisher’s exact test. Associations exhibiting a significantly higher allele frequency in hypersensitive cases (*p* ≤ 0.05) were labeled as SCAR-associated, representing high-confidence positive associations. In contrast, triplets with non-significant differences (*p* > 0.05) or higher allele frequency in controls were labeled as non-SCAR-associated. It is important to note that non-SCAR-associated triplets do not necessarily represent confirmed negative associations, but rather reflect the absence of statistically significant evidence in the available datasets and curated sources. Consequently, this group may include unreported or underpowered positive cases, introducing potential label noise. This limitation should be considered when interpreting model performance and the nature of false positive predictions.

#### 4.1.2. HLA Allele Peptide Processing and Cohort Ethnicity

The HLA allele peptide sequences were obtained from the IPD-IMGT/HLA database [22]. HLA class I peptide-binding domain sequences (α1 and α2 domains) were extracted and preprocessed to preserve biologically meaningful structure. The combined α1–α2 sequences were aligned using MAFFT (v7.526) [23] to ensure positional consistency across alleles. These domains were specifically selected because they form the peptide-binding groove of HLA class I molecules, which is directly involved in antigen presentation and T-cell receptor (TCR) recognition. Variations in these regions are known to influence peptide binding specificity and drug interaction profiles, making them critical determinants of allele-specific drug hypersensitivity reactions. Reference-guided peptide selection was performed using the Research Collaboratory for Structural Bioinformatics Protein Data Bank (RCSB PDB) structure 1A1N as a template, and the aligned residues were encoded into position-specific features. Following multiple sequence alignment, the aligned sequences were segmented into 55 position-specific features, each representing the amino acid residue at a given position. This encoding strategy enables the model to capture residue-level variation relevant to allele-specific drug hypersensitivity. Each site was encoded using position-specific one-hot encoding to represent the observed amino acid residues across alleles. Invariant positions and zero-variance features were removed, resulting in a total of 113 informative HLA sequence-derived features used for downstream modeling.

Cohort ethnicity was derived from population annotations provided in the Allele Frequency Net Database (AFND) [16], which reflect the classifications reported in the original studies. These categories were used directly without additional reclassification or aggregation. Ethnicity was incorporated into the model using one-hot encoding, where each category was represented as a binary feature (e.g., Black, Caucasian, Diverse, and Mongols). Thus, population stratification was modeled at the level of AFND-defined groups rather than broader continental ancestry clusters (e.g., K = 4).

#### 4.1.3. Drug Chemical Structures

Chemical structures for each drug were retrieved from the PubChem database [21] in Simplified Molecular Input Line Entry System (SMILES) format from DrugBank database (https://go.drugbank.com/). These structures were transformed into numerical descriptors using Morgan fingerprints (radius = 2, 1024-bit length), which encode circular molecular substructures into fixed-length binary vectors suitable for machine learning–based SCAR risk prediction. Molecular fingerprints capture structural features relevant to HLA binding and immunogenicity, where subtle variations in chemical substructures may influence antigen presentation and immune activation. The selected configuration (radius = 2, 1024 bits) is widely used in cheminformatics, as it effectively captures local substructures while providing a balance between representational capacity and computational efficiency, with reduced hash collisions. To improve feature quality and reduce redundancy, zero-variance fingerprint features (i.e., bits with no variation across all drugs) were removed. This resulted in a reduced set of 237 informative molecular descriptors, which were used for model training.

#### 4.1.4. Network Construction and Topological Feature Extraction

To capture systemic pharmacological interactions and phenotypic manifestations relevant to ADRs, two complementary biological networks were constructed: a drug–drug interaction (DDI) network and a drug–symptom interaction (DSI) network. In the DDI network, nodes represent drugs and edges represent known drug–drug interactions. In the DSI network, nodes represent drugs and symptoms, and edges represent reported associations between drugs and clinical symptoms. From these networks, topological features including degree centrality and eigenvector centrality were extracted and used as model inputs. These networks enable modeling of both molecular-level interaction structure and clinical-level outcome associations. Drug–drug interaction data were obtained from the DDInter database [24,25] and used to construct an undirected graph in which nodes represent drugs and edges denote known interactions. This network captures pharmacokinetic and pharmacodynamic relationships that may contribute to polypharmacy risk and off-target effects. Drug–symptom interaction data were retrieved from the T-ARDIS database [26,27] and used to build a bipartite graph, where drugs and symptoms form two distinct. This network reflects the phenotypic landscape of drug effects and provides complementary information to molecular interaction data.

For each drug node in both networks, degree centrality and eigenvector centrality were computed using NetworkX version 3.6.1 [28]. Degree centrality quantifies the number of direct connections of a drug, reflecting its interaction burden and potential for polypharmacy-related risk [29]. Drugs with higher degree centrality tend to interact with a larger number of other drugs, which may increase the likelihood of pharmacokinetic or pharmacodynamic interactions and consequently elevate the risk of adverse drug reactions, whereas eigenvector centrality measures the influence of a drug within highly connected subnetworks, capturing its global importance in the interaction topology [30]. These topological features were integrated into the machine learning model to represent the systemic influence of each drug.

### 4.2. Model Training and Evaluation

We applied XGBoost version 3.2.0 for SCAR-associated versus non-SCAR-associated classification. XGBoost classifier was trained using a gradient boosting framework with logistic loss as the optimization objective. Given the pronounced class imbalance, the Synthetic Minority Over-sampling Technique (SMOTE) imblearn version 0.14.1 [2] was applied only within the training partition of each cross-validation fold to improve minority class representation. To prevent data leakage and ensure fair model evaluation, SMOTE was applied only to the training data within each cross-validation fold, while the test data remained completely untouched. While SMOTE improved sensitivity, its use was carefully validated to minimize the generation of biologically implausible samples. All experiments were conducted with a fixed random seed to ensure reproducibility and model implementation was performed using the Scikit-learn library [31] in Python version 3.12.13.

Model training and evaluation were conducted using a nested cross-validation framework. The dataset was divided into 5 folds using stratified sampling to preserve the class distribution of SCAR and non-SCAR samples. In each iteration, one fold (20% of the data) was used as the independent test set, while the remaining four folds (80%) were used for training. Within each training fold, hyperparameter optimization was performed using Optuna 4.8.0 with stratified cross-validation. Within each training fold, SMOTE was applied after the train–test split and before model fitting, while the corresponding test fold remained completely untouched to prevent data leakage.

Model performance was assessed using multiple metrics appropriate for binary classification and imbalanced data. Accuracy represents the proportion of correctly classified instances. Precision evaluates the reliability of SCAR-associated predictions, while recall (sensitivity) measures the ability to correctly identify true ADR cases. The F1-score, defined as the harmonic means of precision and recall, provides a balanced measure of classification performance. These performance measures calculate as follows, where *TP*, *TN*, *FP*, and *FN* denote true positives, true negatives, false positives, and false negatives, respectively.

(1) $$\mathrm{Accuracy}=\frac{\mathrm{TP}+\mathrm{TN}}{\left(\mathrm{TP}+\mathrm{FP}+\mathrm{TN}+\mathrm{FN}\right)}$$

(2) $$\mathrm{Precision}=\frac{\mathrm{TP}}{\left(\mathrm{TP}+\mathrm{FP}\right)}$$

(3) $$\mathrm{Recall}=\frac{\mathrm{TP}}{\left(\mathrm{TP}+\mathrm{FN}\right)}$$

(4) $$F1-\mathrm{score}=\frac{\mathrm{Precision}\times \mathrm{Recall}}{\mathrm{Precision}+\mathrm{Recall}}$$

To further evaluate discrimination ability, we used the Area Under the Receiver Operating Characteristic Curve (AUROC), which quantifies the trade-off between true positive and false positive rates. An ideal classifier achieves an AUROC of 1.0, while random guessing yields 0.5. Given class imbalance, we also reported the Area Under the Precision-Recall Curve (AUPRC), which provides a more informative evaluation when positive cases are underrepresented. AUPRC offers a more sensitive reflection of performance in imbalanced scenarios where positive predictions are more critical. All performance metrics, including AUROC and AUPRC, were computed on the independent test folds in the outer cross-validation loop and averaged across all folds and repeated runs (5 × 10), ensuring an unbiased estimate of the model’s generalizability to unseen data.

## 5. Conclusions and Future Directions

This study presents an integrative, network-informed machine learning framework for predicting HLA-associated severe cutaneous adverse drug reactions (SCARs). By combining HLA sequence features, drug chemical descriptors, demographic information, and interaction network topology, the proposed model addresses key limitations of existing pharmacogenomic approaches, which are largely retrospective or restricted to well-characterized drug–allele pairs. The framework demonstrated strong predictive performance across multiple evaluation metrics. Overall, the final model demonstrated that HLA sequence features and drug molecular fingerprints constitute the most informative feature groups for SCAR prediction, while network-derived and demographic features provide complementary but relatively smaller contributions. This indicates that the predictive signal is primarily driven by genetic and chemical determinants, with additional contextual enhancement from systems-level and population-level features, and exhibited the ability to generalize beyond curated annotations, as evidenced by biologically plausible predictions supported by independent clinical reports.

Notably, several predicted positive associations not present in the training data corresponded to emerging or previously unannotated HLA–drug risk associations, highlighting the model’s potential for discovery and early safety signal detection. This capability underscores the value of integrative, data-driven approaches for uncovering latent immunogenetic risk patterns that may not yet be captured in existing databases.

Despite these encouraging results, several limitations warrant consideration. The dataset size and population composition are constrained by the availability of curated HLA–ADR records, which may limit generalizability across diverse populations. In addition, the use of degree and eigenvector centrality provides only a relatively coarse representation of network topology, and more expressive approaches, such as graph-based embeddings, may further enhance predictive performance. Moreover, while molecular docking offers supportive structural insight, it cannot fully capture the complexity of antigen processing, presentation, and T-cell receptor recognition underlying clinical hypersensitivity reactions.

Future work will focus on expanding the framework to incorporate larger and more diverse pharmacogenomic cohorts, richer chemical and clinical descriptors, and advanced network representation learning techniques. In particular, assembling independent, multi-center datasets will enable external validation and further strengthen the generalizability of the model across different populations and clinical settings. Additionally, integrating structure-informed features and more comprehensive annotation of drug–HLA associations will help reduce prediction uncertainty and improve specificity. An important long-term direction is the development of personalized drug recommendation systems that provide data-driven support for predicting HLA-SCAR risk and suggesting safer therapeutic alternatives when high-risk drug–allele combinations are identified. Such advances have the potential to support precision medicine initiatives, improve drug safety, and reduce the clinical burden of severe adverse drug reactions.

The proposed framework could be integrated into clinical decision-making as part of a pre-treatment screening strategy, where patient-specific HLA typing is performed prior to drug administration and the model is used to estimate SCAR risk. This approach is feasible, as HLA screening is already recommended for certain high-risk drugs, and the model requires minimal computational resources, supporting scalable deployment in clinical workflows. Although implementation involves additional genetic testing costs, these may be offset by preventing severe adverse reactions. However, further validation in large, prospective, and ethnically diverse cohorts is required before routine clinical use.

## Figures and Tables

**Table 1:** Features and their descriptions.

Feature Category	Feature Set	Description
Personal Features	HLA Allele	Protein sequence–derived features of human leukocyte antigen (HLA) molecules, capturing genetic variability associated with immune responses.
	Cohort Ethnicity	Demographic information describing the ethnic background or ancestry composition of individuals within the study cohort.
Drug Features	Drug Structure Data	Chemical structure representations of drugs encoded using Simplified Molecular Input Line Entry System (SMILES), enabling computational extraction of molecular descriptors and properties.
	DDI Network	Topological features derived from the drug–drug interaction network, including degree centrality and eigenvector centrality for each drug.
	DSI Network	Topological features derived from the drug–symptom interaction network, including degree centrality and eigenvector centrality for each drug.

**Table 2:** Summary of performance across validation folds for XGBoost models using different feature sets.

XGBoost with Different Feature Sets	AUROC (Mean ± std)	AUPRC (Mean ± std)	F1-Score (Mean ± std)	Accuracy (Mean ± std)
Baseline (HLA data, Drug structure, Cohort ethnicity)	0.88853 ± 0.03828	0.68953 ± 0.08753	0.58571 ± 0.0740	0.83600 ± 0.03163
Baseline + DSI network	0.88984 ± 0.03791	0.68878 ± 0.08946	0.59715 ± 0.07564	0.83939 ± 0.03015
Baseline + DDI network	0.88896 ± 0.03858	0.68657 ± 0.08989	0.59116 ± 0.07796	0.83867 ± 0.03073
Baseline + DSI network + DDI network	0.88989 ± 0.03771	0.69185 ± 0.08793	0.59155 ± 0.08332	0.83939 ± 0.03201

## Data Availability

All data used in this study are publicly available from the Allele Frequency Net Database (AFND) [16] and the IPD-IMGT/HLA database [22]. The processed data and feature sets generated during the current study are available from the corresponding author upon reasonable request.

## Supplementary Materials

The following supporting information can be downloaded at: https://www.mdpi.com/article/10.3390/ijms27104187/s1.

## Abbreviations

The following abbreviations are used in this manuscript:

AC	Allele Combination
ADRs	Adverse drug reactions
AFND	Allele Frequency Net Database
AUROC	Area Under the Receiver Operating Characteristic Curve
AUPRC	Area Under the Precision–Recall Curve
DDI	Drug–Drug Interaction
DSI	Drug–Symptoms Interaction
EHR	Electronic health record
FN	False Negative
FP	False Positive
HLA	Human leukocyte antigen
MAFFT	Multiple Alignment using Fast Fourier Transform
ML	Machine Learning
RCSB	Research Collaboratory for Structural Bioinformatics Protein Data Bank
ROC	Receiver Operating Characteristic
SCAR	Severe cutaneous adverse drug reaction
SJS	Stevens–Johnson syndrome
SMILES	Simplified Molecular Input Line Entry System
SMOTE	Synthetic Minority Over-sampling Technique
SRS	Spontaneous reporting systems
TCR	T-cell receptor
TEN	Toxic epidermal necrolysis
TN	True Negative
TP	True Positive
XGBoost	Extreme Gradient Boosting
