# Supplementary material for: Integrating Pharmacogenomics and Network Topology for Machine Learning Prediction of HLA-Associated Severe Cutaneous Adverse Drug Reactions
Source: Int J Mol Sci. 2026 May 8;27(10):4187. doi: 10.3390/ijms27104187 (PMC13207861; doi:10.3390/ijms27104187)

Supplementary File S1: Eigenvector centrality and degree distributions

Drug-Drug interaction Network

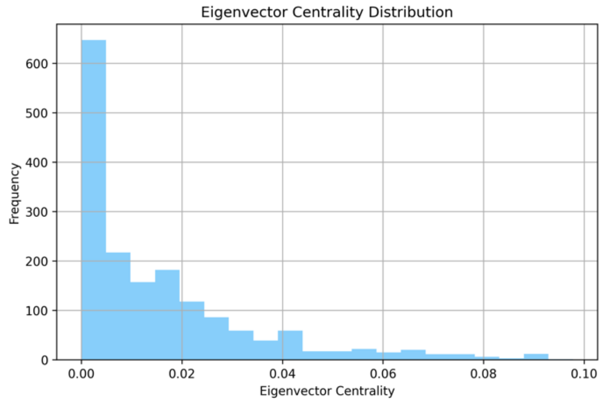

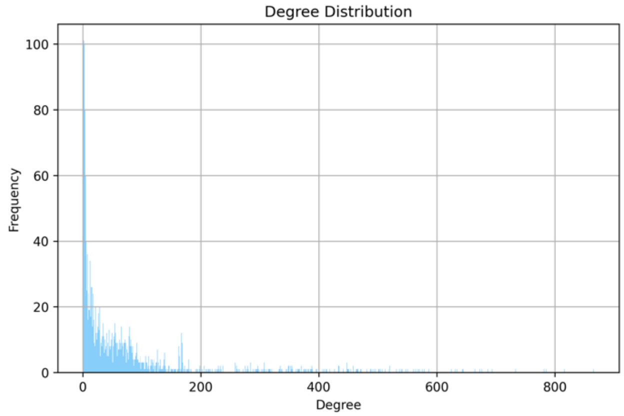

Drug-Symptom Interaction

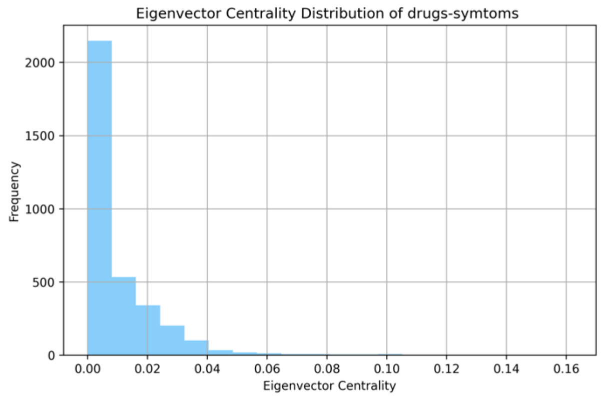

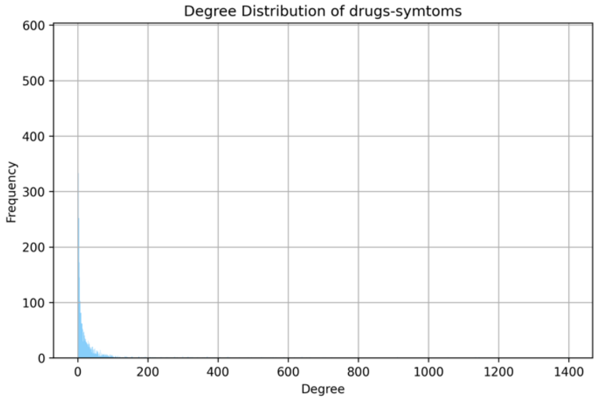

Supplement: Supplementary file 1 [file ijms-27-04187-s001.zip › SupplementaryFileS1.pdf]
